# Computational prediction of actin–actin interaction

**DOI:** 10.1007/s11033-013-2869-8

**Published:** 2013-11-16

**Authors:** Ayhan Ünlü

**Affiliations:** Trakya University Medical Faculty Department of Biophysics, Edirne, 22030 Turkey

**Keywords:** F-actin, G-actin, Protein–protein interaction, Docking, Hot spots

## Abstract

Actin is one of the most abundant proteins in eukaryotic cells, where it plays key roles in cell shape, motility, and regulation. Actin is found in globular (G) and filamentous (F) structure in the cell. The helix of actin occurs as a result of polymerization of monomeric G-actin molecules through sequential rowing, is called F-actin. Recently, the crystal structure of an actin dimer has been reported, which details molecular interface in F-actin. In this study, the computational prediction model of actin and actin complex has been constructed base on the atomic model structure of G-actin. To this end, a docking simulation was carried out using predictive docking tools to obtain modeled structures of the actin–actin complex. Following molecular dynamics refinement, hot spots interactions at the protein interface were identified, that were predicted to contribute substantially to the free energy of binding. These provided a detailed prediction of key amino acid interactions at the protein–protein interface. The obtained model can be used for future experimental and computational studies to draw biological and functional conclusions. Also, the identified interactions will be used for designing next studies to understand the occurrence of F-actin structure.

## Introduction

Actin is a very conserved and plentiful eukaryotic protein. Eukaryotic cells have advanced internal supporting structures which are also known as cytoskeleton. Basically, cytoskeleton structures consist of microtubules, inter-mediate filament and actin filament. Actin filament is found in shapes of globular (G) and filamentous (F) in the cell. The 3D structure of globular (monomeric) actin was first determined by Kabsch et al. [1]. Most functions of actin are regulated by protein–protein interactions. Besides, interacting with an enormously diverse set of other cellular proteins, actin’s most critical functions arise from its interactions with itself as it assembles to form F-actin filaments [2]. Because actin carries out its cellular functions through its filamentous form, knowing the detailed structure of actin filaments is an important step in achieving a mechanistic understanding of actin function [3]. Helix occurs by aligning and polymerization of monomeric G-actin molecules, and is named F-actin. Actin makes up 5 % of all proteins in eukaryotic cells, and about 20 or 25 % of muscle proteins [4]. The primary structure of it contains 375 amino acids, and there are great similarities between species (homology). The primary structure of actin consists of four subunits [5]. The primary subunit includes sequences of 1–32, 70–144, 338–375 amino acids, the second subunit includes sequence of 33–69 amino acids, the third subunit includes sequences of 145–180, 270–337 and the fourth subunit includes sequence of 181–269 amino acids. Each G-actin molecule can ligate an ATP molecule. G-actin molecule may undergo post-synthesis modifications such as acidification from N-terminus and ADP-ribosylation. G-actin molecules can ligate ATP, Ca and Mg [6]. In physiological ionic conditions and in presence of magnesium and ATP, G-actin molecules polymerase ‘un-covalently’ in order to make a couple of helix filaments for F-actin. F-actin is in fibril-structure, 7 mm thick, and 35.5 nm length. About 50 % of actin molecules in animal cells is in monomer-structure. G-actin is in form of free monomer or small complexes with certain proteins. There is a dynamic equilibrium between G-actin and F-actin molecules. This dynamism helps many cellular functions including cell-surface movement to happen [7]. Actin interacts with many cellular proteins besides cytoskeleton and plasma membrane. Eukaryotic elongation factor 1 (eEF1), responsible for synthesis of proteins, eukaryotic elongation factor 2 (eEF2), deoxyribonuclease I (DNase I) involved in apoptosis are some of cellular proteins [8, 9]. In addition, actins of certain bacterial toxins have been reported that they make actin change into ‘ADP-ribose’ [10]. The first structural model of F-actin was obtained by Holmes [11], by using fiber-diffraction data extending to 8.4-Å resolution to determine the approximate orientations and positions of actin protomers in the filament. Despite a general consensus regarding the validity of current models for F-actin, the problem of atomic-level detail remains. The interaction between F-actin and actin-binding proteins such as myosin, cofilin, fimbrin, fascin, villin, a-actinin, etc., is important to so many cellular functions, the lack of a high resolution model of F-actin is a handicap in understanding many of these interactions. Docking is a computational method which predicts the preferred orientation of one molecule to a second when bound to each other to form a stable complex. Knowledge of the preferred orientation may be used to predict the strength of association or binding affinity between two molecules using a scoring function. Docking has been widely used to suggest the binding modes of protein–protein interaction. The growing number of individual structures in the crystallographic databases and the relatively small number of solved complexes has made predictive docking an important theoretical method [12]. Protein interactions are known that a small subset of ‘‘hot spot’’ residues account for most of a protein interface’s free energy of binding. The stability of protein complexes is mediated by a collection of biophysical properties, including hydrophobicity, van der Waals forces, shape specificity, hydrogen bonds, salt bridges, solvent accessibility, and so on [13]. In this study, we attempted to find hot spots between actin and actin interaction using computational prediction and we mapped theoretically determined hot spots and structurally residues to investigate their geometrical organization.

## Materials and methods

### Theoretical calculation of protein–protein interactions

X-ray crystallography or PDB folders formed by Nuclear magnetic resonance (NMR) of primary sequences (patterns) of proteins whose interaction would be determines, were found on www.expasy.org (Expert Protein Analysis System) and www.pdb.org (Protein Data Bank). Academic version of protein analysis softwares such as Pymol, Rasmol were used, and possible interaction surfaces were displayed by mapping related residues of proteins. Proteins, whose patterns were determined before, was put into interaction at ClusPro 2.0 simulation software is readily available on protein–protein docking system at Structural Bioinformatics Laboratories of Boston University. This is protein docking software which is Fast Fourier Transform (FFT) correlation approach, and it has been expanded in order to use double logical interaction potentials. The best 1,000 energy conformations were clustered on this software to be used at possible interactions. First of all, for exploring interaction areas, energy area is widely researched by using a simplified energy model and the theory of restricted flexibility. After, determined areas were focused by using detailed scoring and sampling. Second step of algorithm is a step where clustering of structures for range measurement by using double logic root mean square deviation (RMSD). The biophysical meaning of clustering is to isolate energy basins of highly loaded energy areas. At this software, FFT, which is a docking method with double logic potential applied against PIPER; DARS (Decoys as the Reference State), which is a method to produce reference conditions for molecule identification potentials; a clustering technique for discovering of possible conformations; Semi-Definite programming based Underestimation (SDU) which provides energy optimization and removing of nonlocal clusters by analyzing free energy stability are respectively used [14]. By evaluating ten interaction areas according to thermodynamical energy calculations, areas where possibility of bonding is high, were determined.

There are several programs available for protein–protein docking that attempt to predict the structure of docked complexes when the coordinates of the components are known. In this study, PIPER was selected for performing the docking simulations as it uses Fourier transform to rapidly evaluate the shape complementarities and also it has various post-docking processing methods to score the resultant complexes, including scoring based on electrostatics and experimental data. The predicted actin–actin structure was analyzed with two different visualization programs, namely Swiss-Pdb Viewer and Discovery Studio 3.5, both of which are freely available In Swiss-Pdb Viewer, molecules were superimposed with iterative magic fit tool under fit menu. In Discovery Studio 3.5, molecules were superimposed with fit tool under edit menu. In Swiss-Pdb Viewer, specific amino acid residues were selected from the control panel whenever needed. In Discovery Studio 3.5, selection dialog box was used. The existence of hydrogen bonds was predicted with compute Hbonds tool of Swiss-Pdb Viewer. Ramachandran diagrams were examined via Discovery Studio 3.5. Distribution of electrostatic potentials and temperature factors were calculated in Discovery Studio 3.5. Default values of 1 Å grid step and 4 Å surface-layers were used. Docked complexes were selected and ranked based on a hierarchical clustering method [15]. The individual starting structures for the docking were obtained from the PDB database: the structure of actin (PDB code: 3HBT) both with resolutions of 2.7 Å obtained by X-ray diffraction. The docking run, which results in 10,000 docked complexes, was performed with the inclusion of electrostatic scoring for excluding false positive complexes.

### Scoring and filtering analysis

In order to upgrade these models to reliable predictions, which could be used with confidence for further experimental and computational work, refinement using biological data is done. Docking algorithm attempting to find a complex structure for two given molecules based on surface complementarity and geometric fitting would invariably return several docking poses between the two molecules [16]. The accuracy of the generated actin–actin complexes was further supported by calculating the TM-score [17]. The value of the TM-score for a model was 0.86 which indicated a very fine model prediction as well (for meaningful predictions, TM-score should be bigger than 0.4).

### Molecular dynamics simulations

The best structural model for the complex of actin–actin obtained from the docking procedure was subjected to MD simulation to refine the protein interface. However, no explicit constraint functions were used to maintain the initial docking contacts during the simulation. The structures were first energy minimized using 1,000 steps of steepest descent and 2,000 steps of conjugate gradient minimization using the Kollman all-atom force field implemented [18]. A distance dependent dielectric function was used with the dielectric constant set to 1 and the nonbonded cutoff was set to 8 Å. Energy minimization with classical force field can be used to remove unrealistically close steric clashes and large deviations from ideal geometry resulting from the conformational changes of amino acid side chains after docking, but molecular dynamics simulation is required to improve distributions. This energy minimized structure was used as the starting structure for the MD simulation. All MD simulations were performed with the NAMD (**N**ot (just) **A**nother **M**olecular **D**ynamics program) molecular simulation package. To analyze the binding interactions of between actin and actin molecular dynamics (MD) simulations were performed by using the NAMD version 2.9 at a mean temperature of 300 K and pH 7. NAMD is a parallel molecular dynamics code designed for high-performance simulation of large biomolecular systems. NAMD scales to hundreds of processors on high-end parallel platforms, as well as tens of processors on low-cost commodity clusters, and also runs on individual desktop and laptop computers [19].

The final conformation obtained at the end of the MD simulation was used for identifying specific interactions at the interface, computing inter-residue distances and other calculations.

## Results and discussion

### Modeling of the actin molecules

**Fig. 1:**
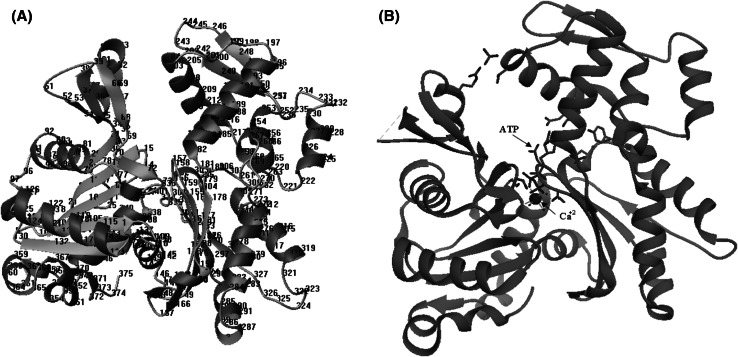
Folding analysis of G-actin **(A)**, binding ATP with Ca^2+^ **(B)**

**Fig. 2:**
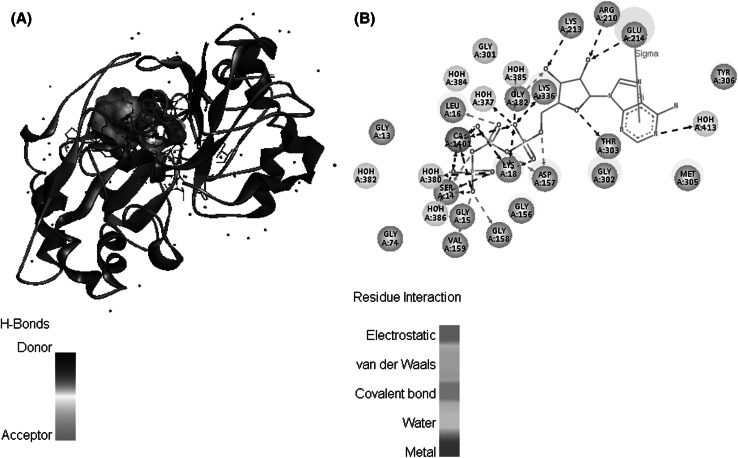
H-Bond analysis of G-actin **(A)**, interaction of actin residue **(B)**

Firstly, the crystal structure of the complex of actin (pdb code 3HBT) was used to build rough model (Fig. 1). Protein structure file (PSF) and protein data bank file (PDB) are archive files of experimentally determined three-dimensional structures of biological macromolecules. The X-ray structure from the PDB file does not contain the hydrogen atoms of ubiquitin. This is because X-ray crystallography usually cannot resolve hydrogen atom. NAMD-compatible PSF and PDB atomic model files had to be generated using PSFGEN. Hydrogens were added to the atoms; in the original pdb file by PSFGEN. The correct protonation state which had already been specified for each of the residues was supplied (Fig. 2).

Actin is a member of a superfamily of ATPases that consist of two domains connected by a hinge, with a nucleotide binding site located in the cleft between the two domains. Transition between a closed and an open state of the nucleotide-binding cleft in G-actin permits nucleotide exchange. The conformational transition between twisted domains in G-actin and a flattened F-actin protomer enhances ATP hydrolysis. In vitro, G-actin is activated with respect to polymerization through the replacement of Ca^2+^ by Mg^2+^ at the ATP-binding site. The smaller radius of Mg^2+^ and its preferred coordination geometry lead to the ejection of one water molecule from the coordination sphere in comparison to Ca^2+^ [20].

### Modeling of the actin–actin complex: protein–protein docking

Predicting protein–protein interactions is inherently challenging owing to the difficulty in modeling the many forces that contribute to these interactions. This leaves the burden of excluding false positives from the docking results and ascertaining whether the model obtained is reliable by using accurate scoring and filtering techniques. To predict formation of a actin–actin complex, ClusPro, an automated docking and discriminating method for prediction of protein complexes, was used via web-based server (http://cluspro.bu.edu/). Docked conformations were generated using the docking program DOT based on FFT correlation approach. Default values of 1 Å gridstep and 4 Å surface-layers were used. Docked complexes were selected and ranked based on a hierarchical clustering method [21]. The structure of actin–actin complex was modelled in ClusPro server.

F-actin is formed by polymerization of G-actin in a process with three distinct stages: activation, nucleation, and elongation [22]. These processes are likely to be accompanied by a number of conformational changes in the actin protomer to allow: the ATP-actin monomer to join the filament, hydrolysis of the ATP, and release of the phosphate. In vitro, metal ion sensitivity, in which Mg^2+^ favors polymerization over Ca^2+^, suggests a fourth conformational change.

**Fig. 3:**
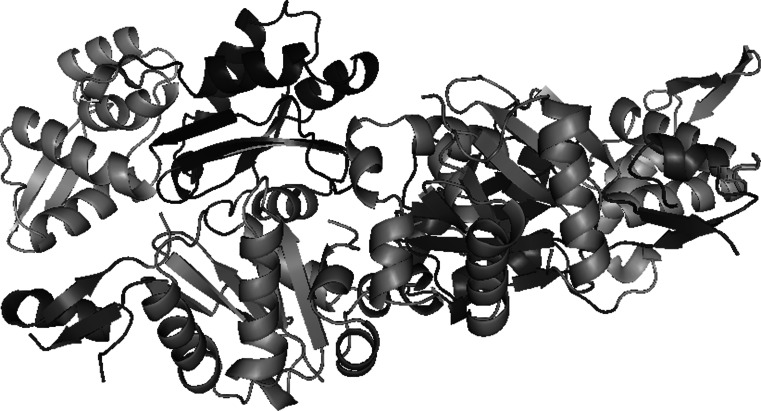
Cartoon representation of the structure of the actin–actin complex obtained through docking simulation. *Red*: subunit 1 (1–32; 70–144; 338–375), *green*: subunit 2 (33–69), *blue*: subunit 3 (145–180; 270–337), *yellow*: subunit 4 (181–269). (Color figure online)

**Fig. 4:**
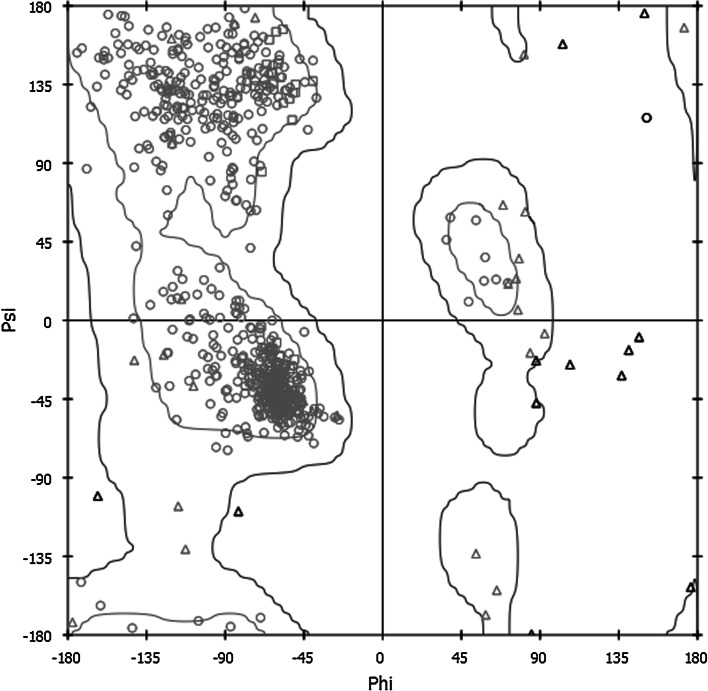
Ramachandran plot for actin–actin model was prepared using Discovery Studio

Apart from using surface complementarity and electrostatic filter, residue pair potentials and biochemical data were also included to score the docking orientations, as it has been shown to produce more accurate results than using geometric fit and electrostatic energy alone. The most favorable solution obtained by this method was then refined through molecular dynamics to get the final docked model (Fig. 3) which was used to analyze the interactions at the protein interface. In addition, each residue has two bonds which can rotate freely. These two angles define the conformation of that residue in a protein and are called the Ramachandran angles, ψ (psi) and φ (phi). Examination of Ramachadran plots of the back bone angle of actin–actin structure model showed that they both fall in the commonly observed regions psi–phi space (Fig. 4).

**Fig. 5:**
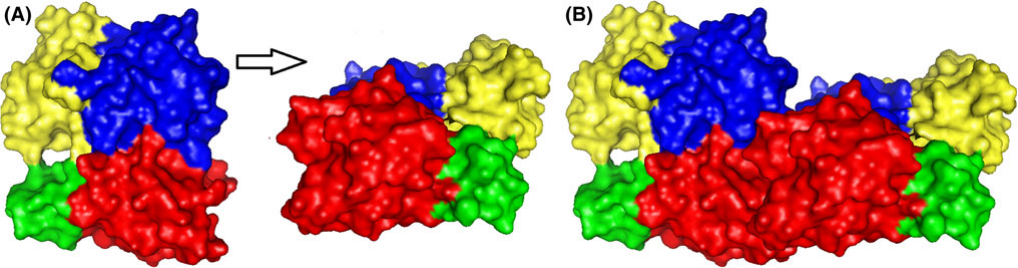
Structure analysis of actin–actin complex drawn using Pymol. *Red*: subunit 1 (1–32; 70–144; 338–375), *green*: subunit 2 (33–69), *blue*: subunit 3 (145–180; 270–337), *yellow*: subunit 4 (181–269). **(A)** Before polymerization **(B)** after polymerization. (Color figure online)

In our study we have identified 3 hot spots that allow the formation of F-actin polymerized G-actin. These are Glu 167, Asp 286, Glu 364 residues. At the end of the docking process we have determined the interactions between Glu 167 and Thr 351; Asp 286 and Ser 350; Glu 364 and Ser 368. We viewed that as a three-dimensional (Fig. 5).

In our efforts to identify the key residues that drive the interaction between actin and actin stabilize their complex, the subunit interactions between the amino acids listed in Table 1 was identified as crucial for binding activity and these binding hot spots will be used to guide interaction identify studies.

**Table 1:** Residues at the interface for actin–actin interactions (range 1.5–4.2 Å**)**

Subunit	Residue	Position	Atom1	Subunit	Residue	Position	Atom2	Distance (Å)
1	GLU	364	OE1	1	SER	368	HG	1.9
1	GLU	364	OE1	1	SER	368	OG	2.8
1	GLU	364	OE2	1	SER	368	HG	2.7
1	GLU	364	OE2	1	SER	368	OG	3.5
1	GLU	364	CD	1	SER	368	HG	2.5
1	GLU	364	CD	1	SER	368	OG	3.5
3	GLU	167	OE1	1	TYR	351	HG1	1.9
3	GLU	167	OE2	1	TYR	351	HG1	4.0
3	GLU	167	OE1	1	TYR	351	CG2	4.2
3	GLU	167	CD	1	TYR	351	HG1	3.2
1	SER	350	CB	3	ASP	286	CG	3.7
1	SER	350	HG	3	ASP	286	OD1	3.0
1	SER	350	OG	3	ASP	286	CG	4.4
1	SER	350	CB	3	ASP	286	OD2	3.2
1	SER	350	HG	3	ASP	286	CG	3.8
1	SER	350	HG	3	ASP	286	OD2	3.9

**Fig. 6:**
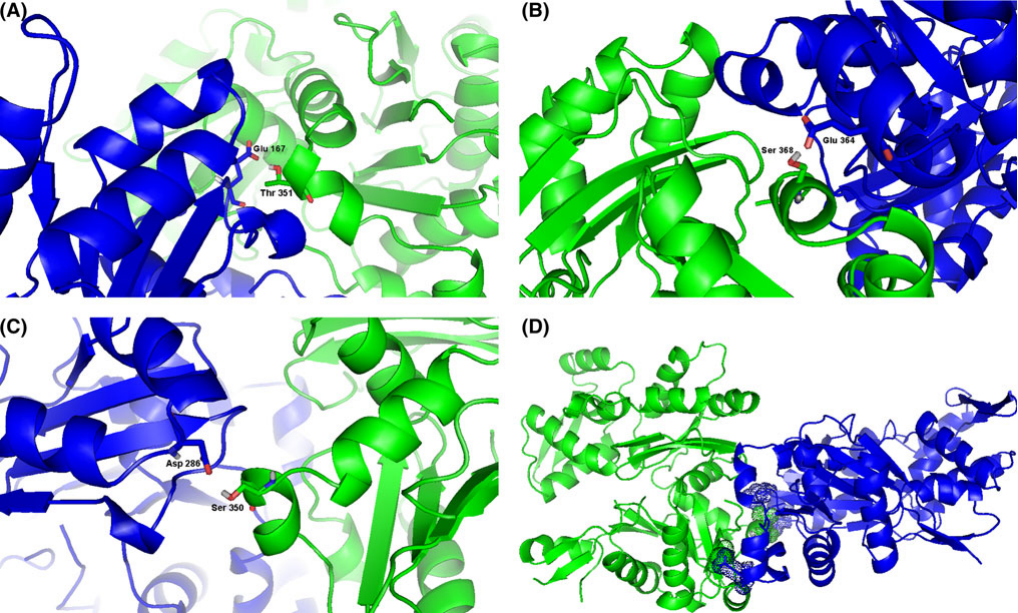
Folding of interaction area between actin–actin complexes. Residues that stabilize complex formation include: **(A)** Glu 167 with Thr 351; **(B)** Glu 364 with Ser 368; **(C)** Asp 286 with Ser 350, **(D)** actin–actin complexes

The interactions involving these residues (Fig. 6) at the interface of actin and actin contribute a large fraction of binding free energy, highlighting their importance in stabilizing the protein complex.

The structure presented here in confirms the twisted conformation of the two domains to be characteristic of monomeric, unmodified G-actin, and that a relative rotation between them must occur to give the flattened conformation observed for an F-actin protomer. But how does delivery of G-actin to the growing end of a filament drive the observed change in the orientation of the two domains? This is not understood, however it has been suggested that such a change happens because it strengthens the interactions between adjacent F-actin protomers within the context of the filament [23]. Formation of an F-actin oligomer of sufficient uniformity to crystallize has eluded our best efforts. However, models, such as that proposed by Holmes et al. [11], permit elaboration and testing of hypotheses about intersubunit interactions within actin filaments, and with agents that stabilize or disrupt those same filaments [24].

**Fig. 7:**
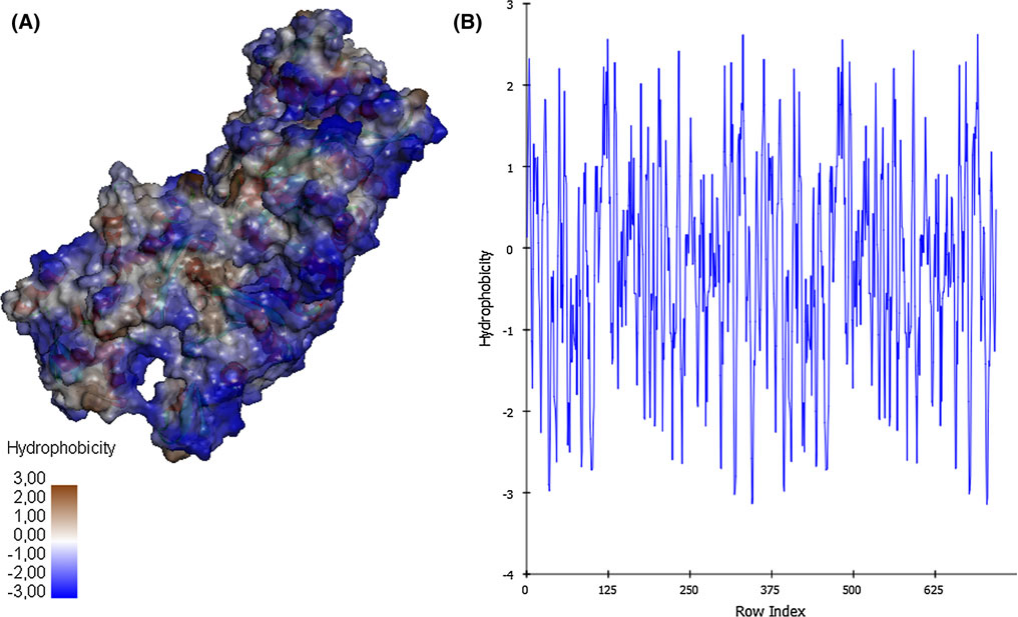
Hydrophobicity of actin–actin complex. **(A)** Hydrophobicity structure analysis of actin, **(B)** hydrophobicity graphical analysis of actin

Proteins have complicated three-dimensional shapes that can include α helices, β sheets and random coil segments. A number of different types of interaction help define the structure. These include hydrogen bonds, electrostatic interactions, van der Waals interactions and hydrophobic interactions. Because proteins fold in an aqueous environment, the contribution of a given interaction to the folding of the protein depends not so much on the strength of interaction within the protein but on the difference between the strength of the interaction within the protein and the strength of interaction of the same groups with water. Hydrophobic interactions, however, are different. As noted above, protein folding occurs in the presence of water and the properties of water are dominated by its propensity to form hydrogen bonds. Polar compounds such as sugars can share hydrogen bonds with water and, for this reason, are readily soluble. In contrast, when a hydrophobic (nonpolar) surface is introduced into an aqueous environment it precludes hydrogen bonding. This preclusion of hydrogen bonding to the hydrophobic surface forces the water molecules to adopt alternative arrangements that permit hydrogen bonding to other water molecules. This imposed restriction on the alignment of the water molecules has an energetic cost and is the physical basis of the hydrophobic effect. Because the folding of a protein includes the removal of many nonpolar side-chains from an aqueous environment and their sequestration from solvent; the energy benefit can be very substantial. As shown in Fig. 7 the hydrophobicities of the given actin–actin complexes vary substantially.

### Molecular dynamic simulation of actin–actin complex

**Fig. 8:**
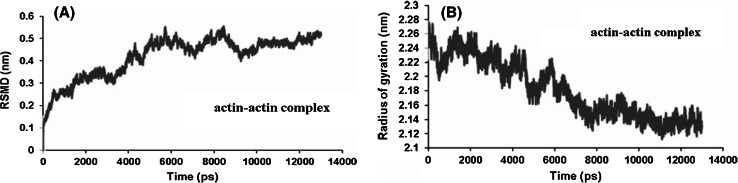
**(A)** RMSD and **(B)** radius of gyration of graph of actin–actin complex

A molecular dynamics simulation of actin–actin complex was performed using the CHARMM force field [25]. The whole simulation experiment was done for 13 ns by using 26.553 water molecules. Docking study between actin and actin revealed significant contribution of hydrogen bonds, attractive van der Waals, repulsive van der Waals, atomic contact energies and global interaction energy of −8.28, −37.23, 16.91, 17.71 and −28.56 (kJ mol^−1^) respectively. The actin–actin complex with the binding energy −27.81 kJ mol^−1^ was further used for carrying out MD. RMSD for all backbone atoms, electrostatic energy, van der Waals energy of actin–actin complex were studied in the form of MD trajectories. RMSD profiles always remained less than 0.5 nm for the entire simulation. The RMSD value for the actin–actin complex increased from 0.059 to 0.38 nm at 3.2 ns, further constantly increased to attain 0.48 nm values at 10 ns and finally attained 0.5 nm around 13 ns depicting a constant RMSD profile during the simulation (Fig. 8a).

The constant trajectory depicted a stabilized complex formation which in turn depicted strong bonding of actin complex. Radius of gyration of actin–actin complex was analyzed to determine its compactness. Radius of gyration value of initial complex configuration is 2.27 nm followed by decrement in value to 2.1 nm around 8 ns (Fig. 8b).

### Computational alanine scanning

The role of individual amino acid side chains in stabilizing the complexes was further probed by computational alanine scanning studies, which identifies residues that are important for the stabilization of the complex, by determining the change in the free energy of binding when various residues in the wild type protein was mutated to alanine [26, 27]. The results from the alanine scanning experiments (Table 2) correlated well with the docking simulation results.

**Table 2:** Result of the predicted contributions of residues through virtual alanine scanning

PDB #	Subunit	Int_ID	ΔΔG (bind)	ΔG (partner)
167	3	1	1.41	−0.38
286	3	1	1.37	0.59
361	1	1	1.46	−0.49
364	1	1	1.23	0.82
351	1	1	1.19	0.06
368	1	1	1.22	−0.75
350	1	1	1.31	1.42

The results from computational alanine scanning confirm that these residues are important for the stability of the complex. Positive values of ΔΔG mean that the alanine mutation is predicted to destabilize the complex and negative values indicate a stabilizing effect. This study indicates that the enthalpic contribution from the desolvation of amino acids, formation of novel H-bonds, van der Waals and electrostatic interactions involving these residues contribute to a favorable free energy of interaction between actin and actin, and offset the decrease in entropy from the loss of translational and rotational degrees of freedom upon binding.

A moderate-sized protein with a molecular weight of 42,000, actin is encoded by a large, highly conserved gene family. Actin arose from a bacterial ancestor and then evolved further as eukaryotic cells became specialized. Some single-celled organisms such as rod-shaped bacteria, yeasts, and amebas have one or two actin genes, whereas many multicellular organisms contain multiple actin genes. For instance, humans have six actin genes, which encode isoforms of the protein, and some plants have more than 60 actin genes, although most are pseudogenes. [28]. Actin exists as a globular monomer called G-actin and as a filamentous polymer called F-actin, which is a linear chain of G-actin subunits. The polymerization of G-actin proceeds in three sequential phases. The first *nucleation phase* is marked by a lag period in which G-actin aggregates into short, unstable oligomers. When the oligomer reaches a certain length, it can act as a stable seed, or nucleus, which in the second *elongation phase* rapidly increases in length by the addition of actin monomers to both of its ends [29]. As F-actin filaments grow, the concentration of G-actin monomers decreases until equilibrium is reached between filaments and monomers. In this third *steady*-*state phase,* G-actin monomers exchange with subunits at the filament ends [30].

As a result of our analysis, we have examined the actin–actin interaction which plays a key role in the process of nucleation phase of actin polymerization by using computerized methods and three hot spots have been identified as Glu 167, Asp 286, Glu 364 residues. At the end of the docking process we have determined the interactions between Glu 167 and Thr 351; Asp 286 and Ser 350; Glu 364 and Ser 368.

## Conclusion

In this study, we have demonstrated the application of protein–protein docking simulation to build a complex structure of actin–actin starting from unbound proteins using the program PIPER. This study is based on the argument that, starting from unbound structures, computer docking simulation can be used to build a set of atomic models of complexes, one of which will be close to the native complex structure, and by applying proper filtering and scoring methods, it is achievable to select the right structure from the docking results. We have utilized electrostatics, residue pair potentials and biochemical information to filter and sort the docked models and build a reliable model of the complex structure. At the end of the filtering process, the final model of the complex was selected that agreed best with the biological data and this model was refined using molecular dynamics, to analyze the interactions and determine hot spot residues. These hot spots at the protein–protein interface, which are small regions that are essential to binding, can be targeted by small molecules to imitate the protein–protein interactions. Hence, by combining biological information with computational docking, we have been able to put forward a model in which actin–actin complex binds. This model can be used for future experimental and computational studies to draw biological and functional conclusions.
